# Arrayed waveguide lens for beam steering

**DOI:** 10.1515/nanoph-2022-0198

**Published:** 2022-08-03

**Authors:** Mostafa Honari-Latifpour, Ali Binaie, Mohammad Amin Eftekhar, Nicholas Madamopoulos, Mohammad-Ali Miri

**Affiliations:** Department of Physics, Queens College of the City University of New York, Queens, 11367, New York, USA; Physics Program, The Graduate Center of the City University of New York, New York, 10016, NY, USA; Microsoft Research, Microsoft Corporation, Seattle, WA, USA; Department of Electrical Engineering, The City College of New York, New York, NY, USA

**Keywords:** beam steering, integrated waveguide lenses, photonic lattices

## Abstract

Integrated planar lenses are critical components for analog optical information processing that enable a wide range of applications including beam steering. Conventional planar lenses require gradient index control which makes their on-chip realization challenging. Here, we introduce a new approach for beam steering by designing an array of coupled waveguides with segmented tails that allow for simultaneously achieving planar lensing and off-chip radiation. The proposed arrayed waveguide lens is built on engineering the evanescent coupling between adjacent channels to realize a photonic lattice with an equi-distant ladder of propagation constants that emulates the continuous parabolic index profile. Through coupled-mode analysis and full-wave numerical simulations, we show that selective excitation of waveguide channels enables beam steering with large field-of-views of ∼60°. The proposed arrayed waveguide lens can serve as a compact component in integrated photonic circuits for applications in imaging, sensing, and metrology.

## 1 Introduction

A planar lens that is compatible with integrated photonic circuits is a key component for analog optical signal processing [1, 2]. Integrated photonic lenses are integral parts of chip-scale beam steering systems based on the focal plane array concept, which has a wide range of applications in imaging, wavefront sensing, and light detection and ranging (LiDAR) [3–7]. In such systems, the lens maps focused light at different spatial points on its focal plane to collimated beams propagating at different spatial angles. Therefore, it enables beam steering without a need for phase shifters, as commonly used in optical phased array systems, which are bulky elements at optical frequencies.

Over the years, many efforts have been devoted to realizing a planar lens, while there has been a particular interest in a two-dimensional Luneburg lens [4, 8], [9], [10], [11], [12], [13], [14]. Luneburg lens is a circularly-symmetric gradient-index lens, which is inherently free of aberrations [15]. However, given that it requires a delicate control over the index profile, a planar implementation of the Luneburg lens has remained challenging. It requires either controlling the height of a bulk dielectric waveguide or segmentation to sub-wavelength inclusions that can effectively mimic a continuous profile. On the other hand, in the recent years despite great progress in realizing free-space metalenses [16–22], the realization of on-chip lenses has remained challenging due to the difficulty of manipulating index control while maintaining lateral confinement. Given the technical challenges of realizing integrated geodesic lenses with gradient index profiles, there is great interest in developing alternative planar designs.

**Figure 1:**
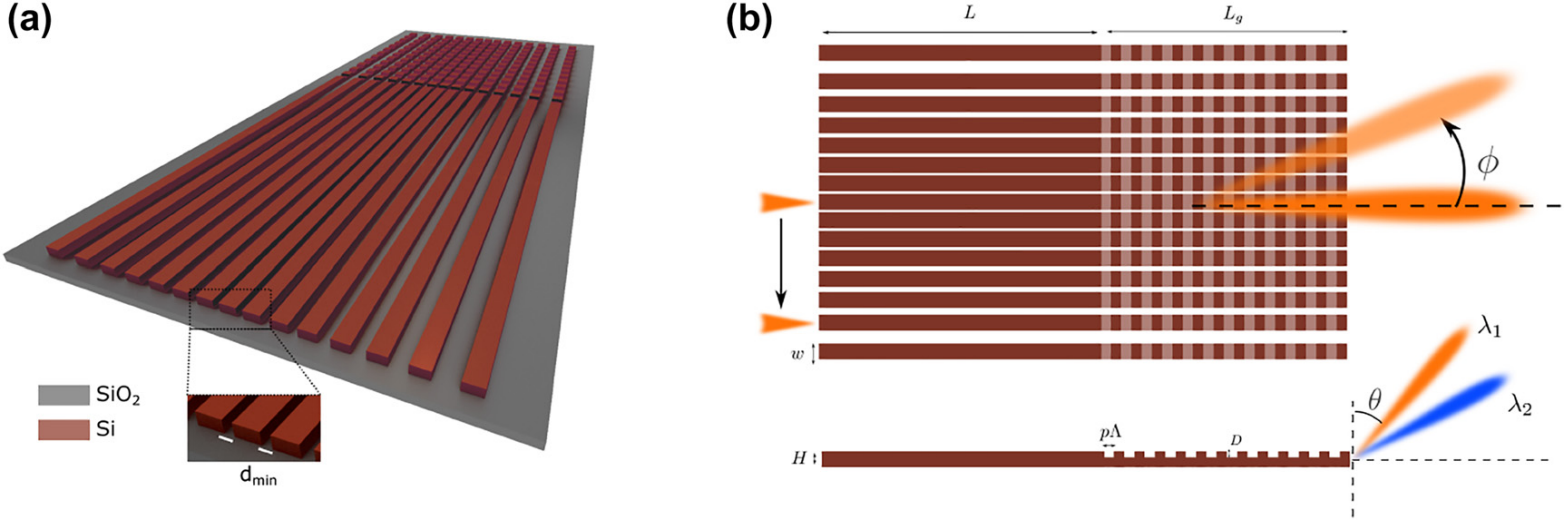
A schematic illustration of beam steering using the proposed waveguide array lens. (a) A scheme of the AWL that is composed of an array of waveguides with nonuniform spacing and structured waveguide tails that create an integrated grating coupler. (b) Beam steering along the azimuthal (*ϕ*) and polar (*θ*) angles is achieved through exciting different channels and changing the excitation wavelength, respectively.

In this paper, we introduce a novel integrated photonic lens, the arrayed waveguide lens (AWL), which is composed of an array of identical parallel waveguides with distributed evanescent coupling, as shown schematically in Figure 1. The proposed lens is inspired by the so-called Jx lattice that due to its particular hopping rates emulates a discrete counterpart of the parabolic potential in quantum mechanics in that it allows uniform spacing of the eigenvalues [23, 24]. Here, we show that this interesting property allows for realizing an integrated photonic lens which enables wide-angle beam steering through selective channel excitation. We provide a systematic design of the arrayed waveguide lens along with an integrated grating coupler based on standard silicon-on-insulator waveguides at telecommunication frequencies. Our results show that beam steering with a field of view of ∼60° can be achieved, while the angle scanning resolution can be controlled with the number of the waveguides involved.

## 2 Results

To describe the arrayed waveguide lens, we first consider a general array of *N* identical waveguides with nearest-neighbor coupling. The propagation of light in the array can be described by a set of first-order equations, d*a*_*i*_/d*z* = *iκ*_*i*,*i*+1_ *a*_*i*+1_ + *iκ*_*i*,*i*−1_ *a*_*i*−1_, where, *i* = 1, … *N* labels the channels, and *z* is the propagation distance [25]. In these relations, *a*_*i*_(*z*) is the complex modal field amplitude at the *i*’th waveguide, and *κ* is the coupling strength between two adjacent waveguides. For a given length *L*, the waveguide array is a multi-input multi-output system that can be described through the transfer matrix relating the fields at its inputs and outputs as:

(1) $$\left[\begin{array}{c} B_{1}\\ B_{2}\\ :\\ B_{N} \end{array}\right]=\left[\begin{array}{cccc} u_{11} & u_{12} & .. & u_{1N}\\ u_{21} & u_{22} & .. & u_{2N}\\ : & : & . & :\\ u_{N1} & u_{N2} & .. & u_{NN} \end{array}\right]\left[\begin{array}{c} A_{1}\\ A_{2}\\ :\\ A_{N} \end{array}\right].$$

Here, *A*_*i*_ = *a*_*i*_(0) and *B*_*i*_ = *a*_*i*_(*L*) (*i* = 1, … *N*) represent the field amplitudes at the inputs and outputs of the system respectively, and *u*_*ij*_ is a complex number that projects the input at port *j* to the port *i* at the output. The transfer matrix can be written as *U* = exp(*iHL*), where *H* is a tight-binding Hamiltonian with off-diagonal elements *h*_*i*,*i*+1_ = *h*_*i*+1,*i*_ = *κ*_*i*,*i*+1_, while all other matrix elements are zero.

Considering a general waveguide array with *N* identical channels, one has *N* degrees of freedom to manipulate the matrix elements *u*_*ij*_ for performing the lensing task. These involve *N* − 1 coupling coefficients and the length of the lattice *L*. Therefore, one can treat this as an inverse problem to find the optimal set of parameters for a waveguide array to operate as a lens. This can be formulated as finding the array parameters for the array that projects shifted discrete delta functions at the input to discrete plane waves with different phase tilts at the output, which, in essence, is a discrete Fourier transform. However, here we take an alternative route and take advantage of a known lattice model, called the Jx lattice. The Jx lattice has been previously introduced in photonics as an interesting platform for perfect state transfer [23, 24], and for performing discrete fractional Fourier transform [26, 27]. Carrying its name from the angular momentum operator $J_{x}$ [28], the Jx lattice is known to exhibit a ladder of equi-distant eigenvalues. It involves symmetrically distributed hopping rates [23].

(2) $$\kappa_{i,i+1}=\frac{\kappa_{0}}{2}\sqrt{\left(N-i\right)i}\;\;\;;\;\;\;i=1,2,\ldots ,N-1$$

where, *κ*_0_ is a scaling parameter for all the coupling coefficients. For an array of identical single-mode waveguides with propagation constant *β*_0_ and with coupling coefficients of relation (2), the propagation constants of the supermodes are found to be equi-distant and given by *β*_*m*_ = *β*_0_ + *mκ*_0_ for *m* = −*N*/2, … *N*/2.

The equal eigenvalue spacing results in self-imaging, i.e., after certain propagation length, *z*_*i*_, a discrete wave profile is reconstructed. This interesting property can be explained readily as follows. Considering a given initial excitation, say **a**(*z* = 0) = **a**_0_, the field profile in the lattice at all propagation distances can be written in terms of the lattice eigenmodes according to **a**(*z*) = ∑_*m*_ *α*_*m*_ **v**_*m*_ exp(*iβ*_*m*_ *z*), where $\alpha_{m}=v_{m}^{†}a_{0}$ are projections of the excitation field on the eigenmodes. Given *β* = *β*_0_ + *mκ*_0_, the field along the lattice becomes **a**(*z*) = exp(*iβ*_0_ *z*)∑_*m*_ *α*_*m*_ **v**_*m*_ exp(*imκ*_0_ *z*). This can be written as **a**(*z*) = exp(*iβ*_0_ *z*)**u**(*z*), where **u**(*z*) = ∑_*m*_ *α*_*m*_ **v**_*m*_ exp(*imκ*_0_ *z*) is clearly a periodic function of *z*, i.e., **u**(*z* + *z*_*i*_) = **u**(*z*) with the self-imaging distance of *z*_*i*_ = *π*/*κ*_0_.

For a single channel excitation, one can show that the discrete beam reaches a maximum width at half of the self-imaging length. At this length, the wavefront contains a phase tilt that is proportional to the location of the excitation channel. These two conditions provide all the requirements for emulating an integrated lens. Thus, in order to perform discrete lensing, the length of the array is taken as half of the revival length, i.e.,

(3) $$L=\pi /\left(2\kappa_{0}\right)$$

which, can be considered as the focal distance of the arrayed waveguide lens. In the following, first, we numerically investigate the AWL for in-plane radiation. Next, we discuss the design of a three-dimensional array with an embedded diffraction grating for beam steering.

**Figure 2:**
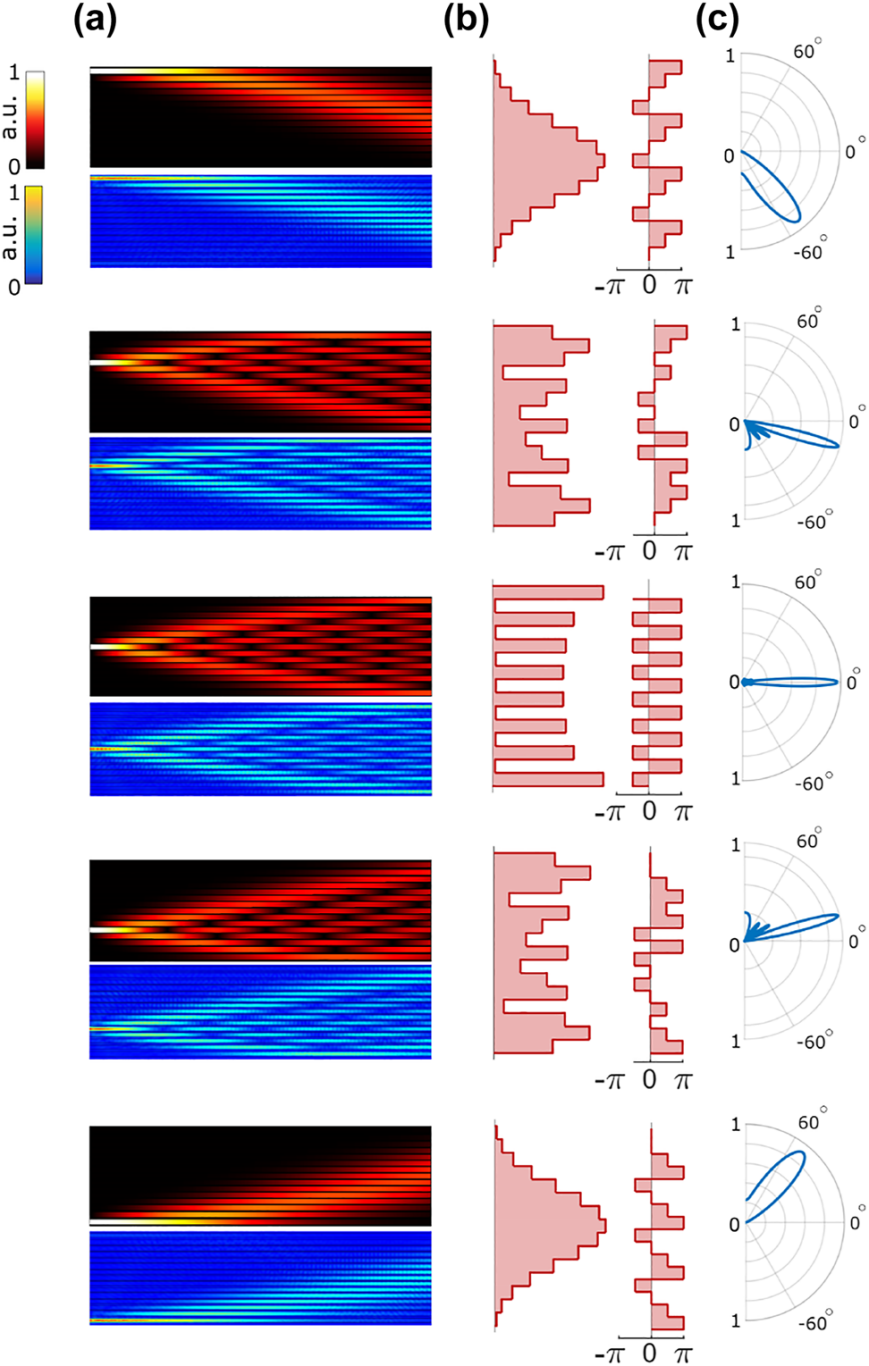
Exciting the waveguide array through different channels. (a) The longitudinal evolution of the field amplitudes through the waveguide lens. In each case, the results in the top panels are obtained through coupled-mode theory, while the bottom panels show two-dimensional finite-element simulations. (b) The magnitude and phase of the modal amplitudes at the output of the waveguide array. (c) The in-plane far-field radiation intensity patterns shown in polar plots with a linear radial axis.

Figure 2(a) depict the evolution of light in the AWL, with coupling coefficients described in relation (2), and composed of *N* = 15 waveguides for different channel excitations. In these figures, the hot- and cold-themed colormaps, respectively, represent the results from coupled-mode theory and rigorous two-dimensional finite-element simulations, which show perfect agreement. For 2D simulations, we utilized the effective index method for an array of silicon-on-insulator (SOI) ridge waveguides at the telecommunication wavelength (*λ*_0_ = 1550 nm). In this case, the coupling coefficients of relation (2) were implemented by engineering the distance between adjacent channels. The details can be found in the Supplementary Information. As shown in Figure 2(b), the designed array suitably projects the input signal to the output ports with desirable amplitudes and phases, that enables beam steering to a set of discrete in-plane angles.

Figure 2(c) shows the associated in-plane far-field radiation patterns. The radiation patterns are obtained by using the array factor *AF* [29], which for single channel excitation at site *j* can be written in terms of the lens transfer matrix as $AF_{j}=\sum_{i=1}^{N}u_{ij}\exp\left(-ik\cdot r_{i}\right)$ , where **k** and **r**_*i*_ show the in-plane wavevector and the position vector of the waveguide outputs, respectively.

It is important to note that the transmission matrix elements of an array of coupled identical waveguides take quaternary discrete phase values of *mπ*/2, where *m* is an integer [30]. Therefore, single-channel input excitation results in quaternary discrete phase values at the output. On the other hand, in an emitter array, the beam steering efficiency is related to the number of available phase steps *q* according to *η* = [sin(*π*/*q*)/(*π*/*q*)]^2^, which fundamentally limits the beam steering efficiency of a waveguide array lens to *η* = 0.81. This constraint on the phase steps at the array output (Figure 2(b)) manifests itself in the existence of sidelobes in the far-field pattern. In addition, while the beam emission direction hops at uniform angles, we observe a jump in the emission angle when exciting the ports located at the edges (Figure 2(c)). Although one can bypass this issue simply by excluding excitation from this port, as we discuss later multi-input excitation can also be utilized to circumvent this problem. According to (1), multi-input excitation not only improves the minimum resultant phase steps, but also provides a tapered amplitude distribution at the output of the lattice, which is known to improve beam steering.

To justify the operation of the proposed structure, we provide a realistic design on a CMOS compatible SOI platform, with 2um buried oxide. In doing so, first, we design the proposed arrayed waveguide lens with an array of ridge waveguides with length *L*. Next, the channels are extended for an extra length *L*_g_, and the extension of each channel is segmented to create an integrated grating coupler as shown in Figure 1(b). The entire structure is simulated with FDTD to investigate its off-chip radiation and beam steering functionality.

In this exemplary design, we consider an array of 15 standard SOI ridge waveguides of width *w* = 400 nm and height *h* = 220 nm, and design the system at the telecommunication wavelength *λ*_0_ = 1550 nm for the fundamental TE mode. The desired couplings between adjacent waveguides (Eq. (2)) can be translated into different waveguide separation distances. It is worth noting that in this system the smallest separation between two channels that is associated with the strongest possible coupling, is a design degree of freedom. A lower bound on the smallest gap between the channels is set by the minimum fabrication size assigned by typical silicon photonic manufacturing processes. To ensure its feasibility with standard fabrication techniques, a lower bound of 70 nm can be considered for the minimum edge-to-edge separation between two channels [31]. Here, by using 100 nm as the minimum separation distance, the maximum coupling value is obtained, and according to Eq. (2) all other coupling values and the separations between all the waveguides are calculated (see the Supplementary Information for details). In this design, the length of the lens, which also scales with the minimum distance between the adjacent channels, turns out to be *L* = 29.5 um.

In the next step, a compatible grating coupler which contains an array of segmented waveguides is designed. The waveguides are extended from the lens and follow the same geometry (400 nm width, 220 nm height), while the grating is formed by considering periodic etching of the tail of the waveguides with a *p* = 50% duty cycle and a *D* = 70 nm etching depth. These parameters result in an off-chip radiation angle of *θ* = 17° (with respect to the normal) at the central wavelength of *λ*_0_ = 1550 nm. In our simulations, we used a total of 13 unit cells with a pitch of Λ = 0.9 um in each waveguide, which result in a total grating length of *L*_g_ = 11.7 um for the integrated grating coupler. It is important to note that the grating coupler follows the transverse geometry of the lens, which inevitably results in transverse coupling of light between channels in the grating section. Thus, the guided beam will follow the dynamics forced by the transverse array profile toward reaching a focus within the grating section, which can adversely affect the radiation pattern. However, this issue can be circumvented by a proper design of the grating coupler to guarantee efficient off-chip radiation in a relatively short length. This aspect is considered in the design described above.

**Figure 3:**
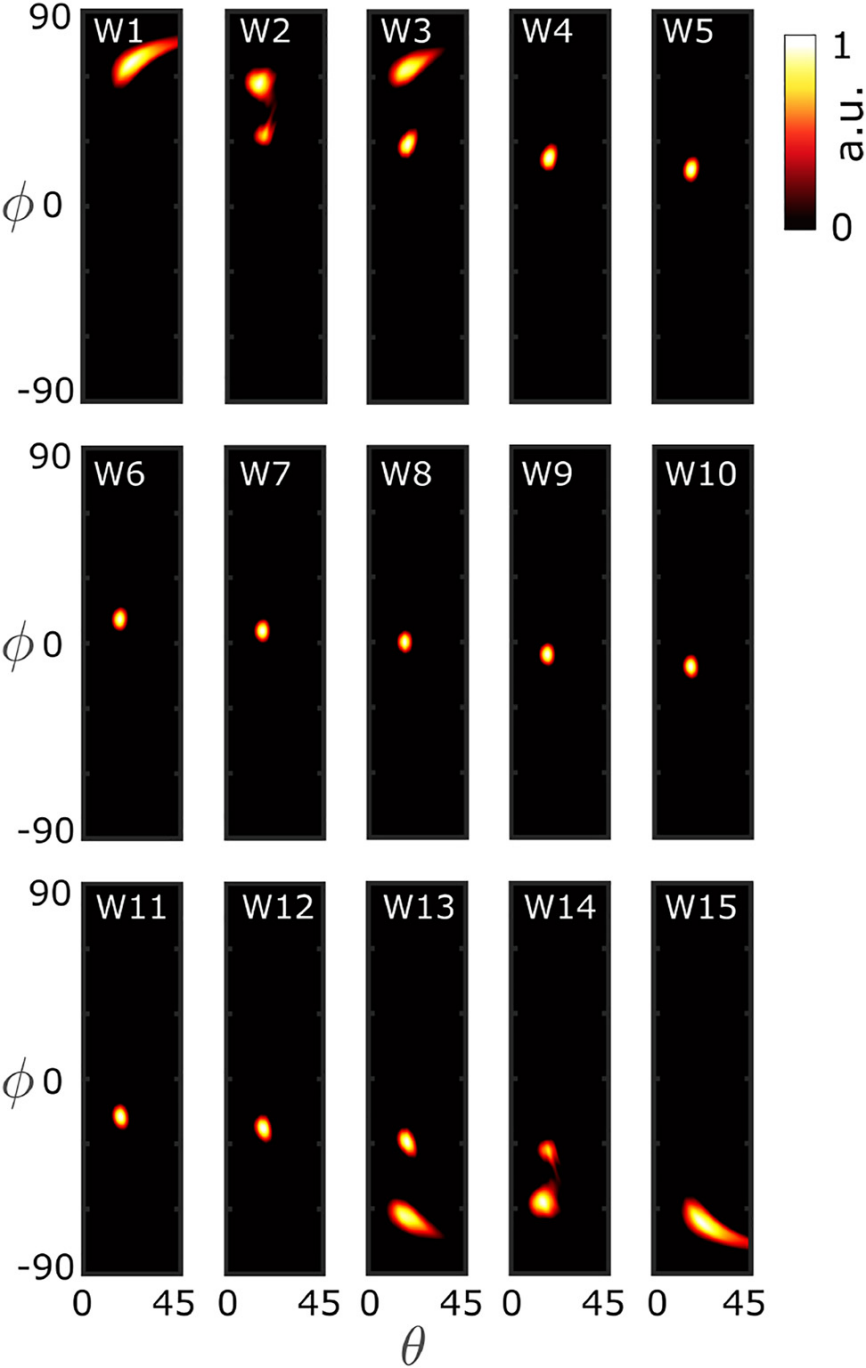
Simulated results of the radiated far-field beam intensity profile of a 15-channel arrayed waveguide lens when excited from different sites. Here, we considered an array of 15 standard SOI ridge waveguides of width *w* = 400 nm and height *h* = 220 nm, at the telecommunication wavelength *λ*_0_ = 1550 nm. The designed integrated grating coupler has a pitch of Λ = 0.9 um, and is composed of 13 unit cells. In each panel the beam intensity is normalized to its maximum value.

We performed a three-dimensional full-wave finite-difference time-domain (FDTD) simulation of the designed structure. Figure 3 shows the radiated far-field beam profiles for different input excitation ports. As it can be seen, by changing the port of excitation the propagation discretion of the beam scans the azimuthal angle *ϕ*. For the reasons discussed before, exciting the ports 2, 3, (and symmetrically ports 13, and 14) result in large undesirable sidelobes. To bypass this issue, we explored mutliport excitation.

**Figure 4: j_nanoph-2022-0198_fig_004:**
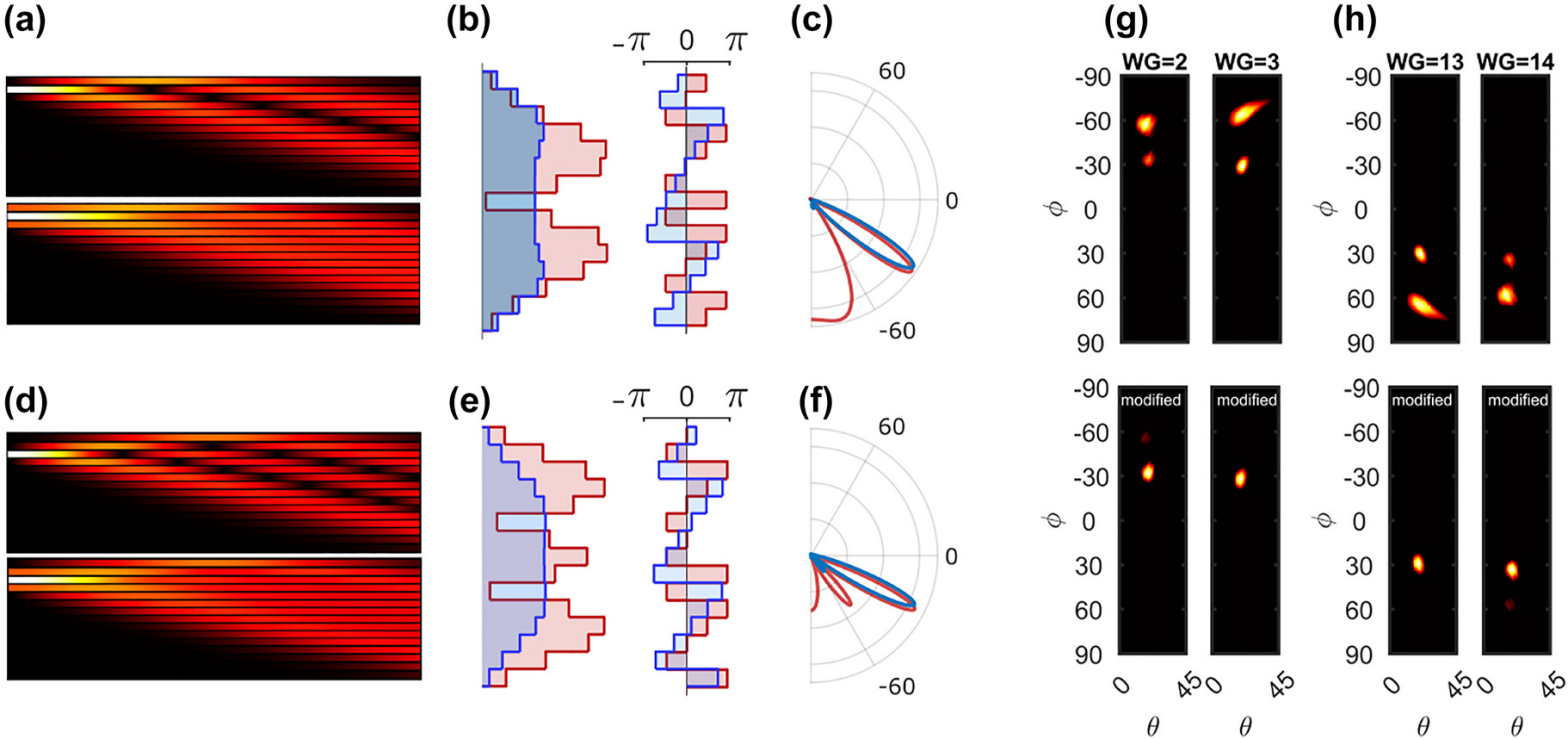
A comparison of the single-versus multiple-channel excitation when exciting ports 14 (a–c) and 13 (d–f) of an array of 15 elements. (a & d) The evolution of the electric field amplitudes along the array. (b & e) The output amplitude and phase patterns. (b & e) The in-plane far-field radiation patterns. In (c & f) red and blue correspond to single- and multiple-channel excitation, respectively. (g & h) The radiated far-field beam profile when the array is excited from ports 2, 3 (g) and 13, 14 (h) with single- (top) versus triple-port excitation scenarios (bottom). In the bottom panels, for excitation with a given port, the neighboring channels are excited with amplitude ratios of 0.5 with respect to the target channel. In each panel of (g & h) the beam intensity is normalized to its maximum value.

Figure 4 compares the single-versus multiple-channel excitation when the exciting ports are located near the edge of the array. As this figure indicates, when only one channel, located near the edge of the array, is excited, a relatively large sidelobe exists. However, simultaneous excitation of adjacent channels, with a profile that approximates a discrete Gaussian intensity distribution (*A*_*i*−1_, *A*_*i*_, *A*_*i*+1_) = (0.5, 1, 0.5) can suppress this unwanted sidelobe. The resulting output phase profiles show a favorable gradual change and a smooth amplitude distribution that result in sidelobe suppression in the spatial beam compared to the case with single-input excitation. It is worth mentioning that using triple-input excitation improves the sidelobe rejections for other cases as well. Figure 4(g and h) shows off-chip radiation for the case with three input excitation ports. In this case, again we observe that 3-port excitation significantly reduces sidelobes when exciting the array from ports near the edges. In addition, it ensures uniform scanning angle hopping when changing the excitation channel.

**Figure 5:**
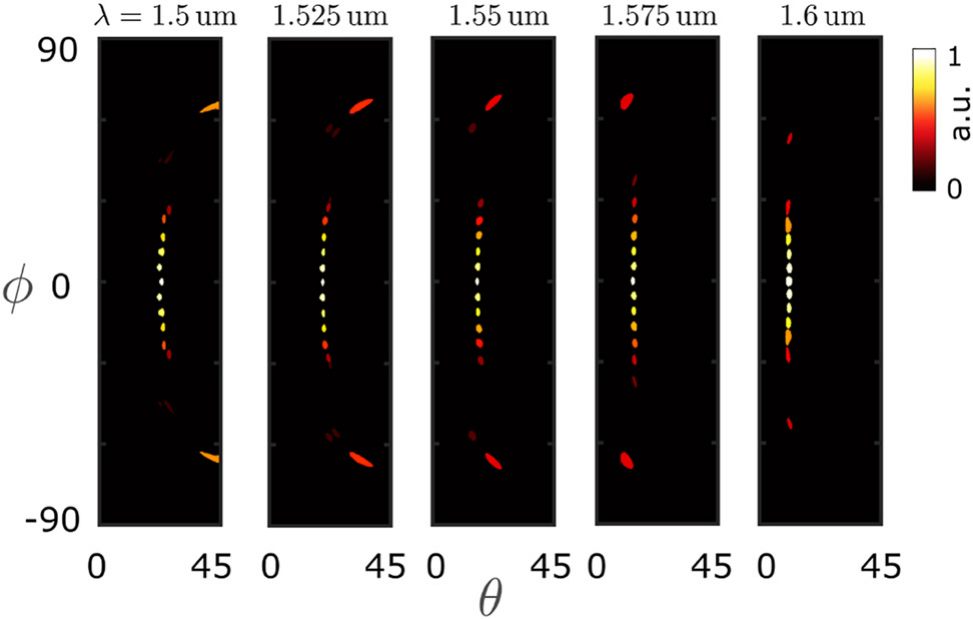
The radiated far-field beam profile of 15-waveguide array at 5 different wavelengths, demonstrating the potential for polar angle beam scanning through wavelength tuning. In each panel all beam intensities are normalized to the peak intensity of the central beam.

The grating coupler allows for scanning the beam direction along the polar angle by tuning the operating wavelength. Figure 5 shows the simulation results of the previous design for different input excitation ports and several different wavelengths. According to this figure, by changing the excitation port, from port 1 to 15, the azimuthal angle of radiation *ϕ* changes in a ∼60° range. To steer the beam in polar angle *θ*, the wavelength is changed from 1500 nm to 1600 nm which provides ∼15° of tuning range.

## 3 Discussion

It is worth mentioning that the results shown in Figures 3 [Fig j_nanoph-2022-0198_fig_004]–5 are based on an uniform grating coupler. On the other hand, it is known that due to the exponential decay of intensity in an uniform grating coupler, the off-chip radiation efficiency is limited to a theoretical bound of ∼80% [32]. In principle, the efficiency of the grating coupler can be enhanced by chirping the grating pitch [33–35] or through inverse design of more involved structures such as utilizing bilayer guides or substrate reflectors [36–39]. In the proposed arrayed waveguide grating, similar methods can be utilized to optimize the radiation efficiency of a waveguide channel as a building block of the device.

**Figure 6:**
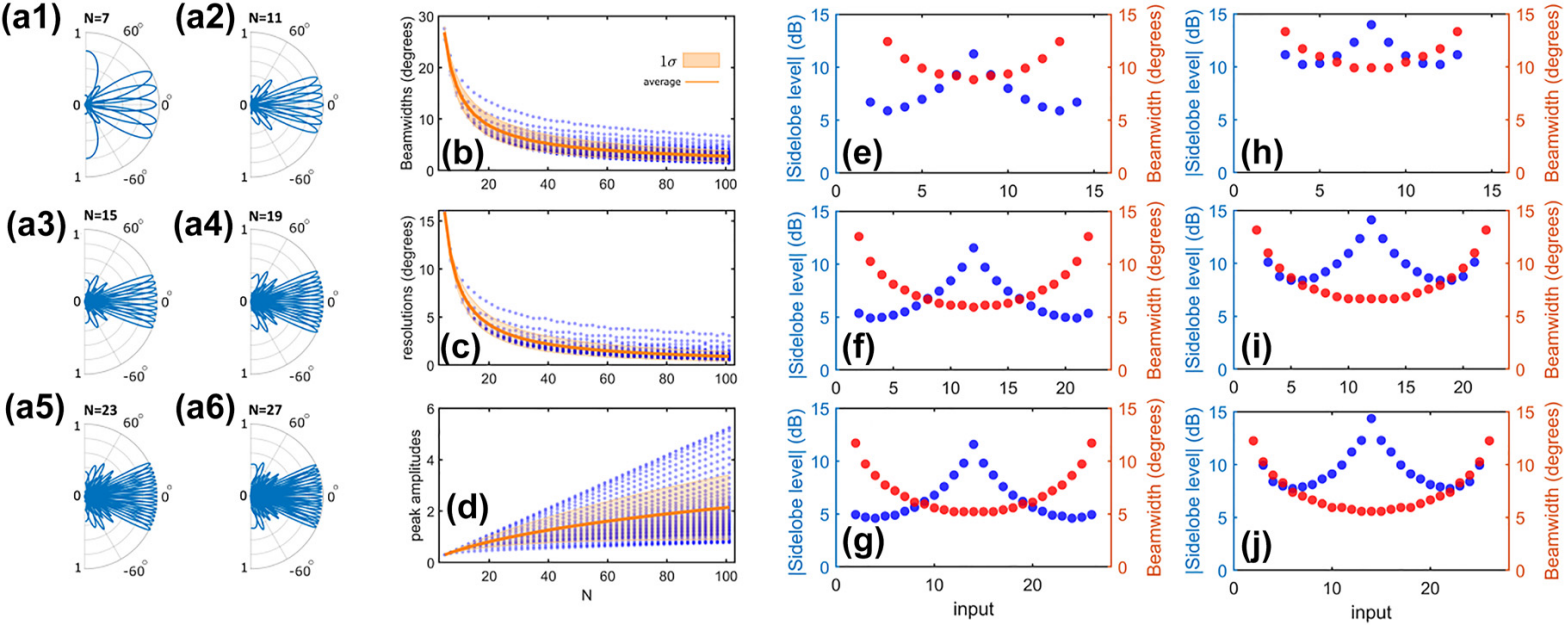
The scaling of the beam steering characteristics with the number of waveguides *N*. (a1–a6) The array factor for *N* = 7, 11, 15, 19, 23 and 27, for excitation from all channels, excluding the peripheral ones. (b, c) The scaling of the (b) beamwidth, (c) angle scanning resolution, and (d) beam peak amplitude with the size of the array. In (b, c), each dot represents excitation from one of the channels. (e, g) Sidelobe level and beamwidth for different excitation channels for array sizes of *N* = 15, 23, and 27. (h–j) Sidelobe level and beamwidth for different excitation channels when using multi-channel excitation for array sizes of *N* = 15, 23, and 27.

The characteristics of the AWL depend on the number of waveguides in the array. Similar to an antenna array, the beam steering performance including the beam quality and resolution are expected to improve by increasing the array size. Figure 6 shows the scaling properties of the arrayed waveguide lens versus the number of channels. According to Figure 6(a1–a6), the field-of-view remains independent from the number of channels. On the other hand, according to Figure 6(b–d) by increasing the number of waveguides *N*, the beamwidths decrease, the scanning angle resolutions improve, and the peak beam amplitudes increase monotonically. Furthermore, Figure 6(d) indicates that the maximum peak amplitude scales linearly with the size of the array *N*, or equivalently the peak intensity scales with *N* ^2^, which shows the superradiance property [40].

We further explored the beam parameters versus the excitation channel for different array sizes. The results are shown in Figure 6(e–g) for array sizes of *N* = 15, 23, and 27, respectively. As these figures show, the beam is optimal when the center channel is excited, with the beamwidth being the smallest and the sidelobe level being the highest. Here, we define the sidelobe level as the ratio of the peak intensity of the main lobe over that of the largest sidelobe. The beamwidth gradually increases and the sidelobe level falls when the excitation is moved toward the peripheral channels. Figure 6(h–j) depict the beam parameters versus excitation channel when considering multi-channel excitation as discussed before. According to these figures, multi-channel excitation significantly improves the sidelobe level while its impact on the beamwidth is negligible.

Finally, it is important to estimate the scaling of the size of the AWL with the number of waveguide channels. Clearly, the total transverse length is proportional with the number of channels *N*. On the other hand, according to relation (3) the array length is also linearly proportional with the number of the waveguides *N*. To better represent this relationship, one can reparametrize relation (2) in terms of the maximum coupling coefficient *κ*_m_, given that the maximum coupling value is explicitly related with the fundamental fabrication and geometric constraints. After a simple reparametrization, relation (2) can be written as $\kappa_{i,i+1}=2\kappa_{\text{m}}\sqrt{\left(N-i\right)i}/\sqrt{N^{2}-1}$ for odd values of *N*, and as $\kappa_{i,i+1}=2\kappa_{\text{m}}\sqrt{\left(N-i\right)i}/N$ for even values of *N*. Accordingly, Eq. (3) can be rewritten as *L* = (*π*/8*κ*_max_)*N* for even number of channels, and $L=\left(\pi /8\kappa_{\max}\right)\sqrt{N^{2}-1}$ for odd numbers, which clearly shows the linear scaling of the length of the AWL with the number of the array elements in both cases.

In summary, we introduced a new integrated lens that is composed solely of an array of evanescently-coupled waveguides with unequal spacing between adjacent channels. The proposed system can be implemented with standard fabrication techniques and does not require spatial control of the refractive index as in other designs based on gradient index materials. The simulation results demonstrated optical beam steering of 60° field-of-view. The proposed lens can serve as an integral component of integrated focal plane arrays for various applications in optical detection and ranging and sensing.

## 4 Materials and methods

The theoretical analysis was built on coupled mode theory. In order to match the parameters of the coupled mode model, we used finite element simulations for finding the propagation constants of the supermodes of two coupled waveguides *β*_e_ and *β*_*o*_. The coupling coefficients were calculated as *κ* = (*β*_e_ − *β*_o_)/2. The array design process for creating the coupling coefficients of Eq. (2) is described in the Supplementary Information. The coupled mode formalism was used for simulating the propagation of light in the array (upper panels in Figure 2(a)), the phase and amplitude distributions at the array output (Figure 2(b)), and the array factors (Figure 2(c)). In calculating the array factor *AF* using coupled mode theory, we assumed uniform spacing between the waveguides since the effect of the actual nonuniform spacings was found to be negligible on the array factor. To justify the results of coupled mode theory, rigorous 2D simulations were performed using finite elements method (FEM) (lower panels in Figure 2(a)). For 2D simulations we utilized effective index method and considered the TE polarization. In 2D simulations, the effective index of the background medium was considered to be *n*_b_ = 1 and the in-plane effective index of the waveguides was considered to be *n*_c_ = 2.7. The patterns in the lower panels of Figure 2(a) show the magnitude of the out-of-plane magnetic field component. The results from the coupled mode model were found to be in excellent agreement with the finite element simulations given that tight confinement of waveguide modes to individual channels justifies the accuracy of the coupled mode theory and neglecting next-nearest-neighbor coupling in this system.

For calculating the far-field beam radiation patterns in Figures 3 [Fig j_nanoph-2022-0198_fig_004]–5, 3D simulations were performed with full-wave finite-difference time-domain (FDTD) method. In these simulations, the refractive index of Si was considered to be 3.47 and the index of SiO_2_ was considered 1.44. The waveguides were excited using their fundamental TE mode. The simulations were performed in a cuboid with open boundary conditions to avoid unwanted reflections. The far-field beam profiles were calculated using the near-field data on the boundaries of the simulation box.

## Supplementary Material

Supplementary Material Details
