# Supplementary material for: Arrayed waveguide lens for beam steering
Source: Nanophotonics. 2022 Aug 3;11(16):3679–86. doi: 10.1515/nanoph-2022-0198 (PMC11501650; doi:10.1515/nanoph-2022-0198)
Supplement: Supplementary file 1 — Supplementary Material Details [file j_nanoph-2022-0198_suppl.pdf]

Arrayed Waveguide Lens for Beam Steering

1. SUPPLEMENTARY NOTE S1: REALIZATION OF THE COUPLING COEFFICIENTS BASED ON RELATION (3) FOR ADJACENT WAVEGUIDES IN THE ARRAY

To realize the coupling coefficients based on the equation (3), first, we consider the propagation of light in coupled waveguides to evaluate their coupling as a function of waveguide separation. We considered two Si waveguides, each with 400μm width and 220μm height built on a SiO_2 substrate and clad with air.

Figure S1 depicts the simulated coupling coefficient for the two coupled waveguides plotted as a function of their edge-to-edge separation d. In this figure, different colors show the coupling for different wavelengths. In the design process, we use this curve to determine the distances between every two adjacent channels such that the couplings are mapped onto the desired relation (Eq. (3) of the main text). In this relation, for $N = 15$, the maximum coupling is for the coupled waveguides in the center of the structure ($i = 7, 8$ and symmetrically $i = 8, 9$), which corresponds to the minimum waveguide separation, i.e. 100nm in our design. Relation (3) of the main text defines all other coupling coefficients, from which, by utilizing Fig. S1, we can translate the coupling factors to waveguide separations. Table S1, shows the waveguide-separation for different coupled waveguides.

Table S1. Waveguide separation for different coupled waveguides

WGs	(1&2) (14&15)	(2&3) (13&14)	(3&4) (12&13)	(4&5) (11&12)	(5&6) (10&11)	(6&7) (9&10)	(7&8) (8&9)
d (nm)	181	143	124	113	106	102	100

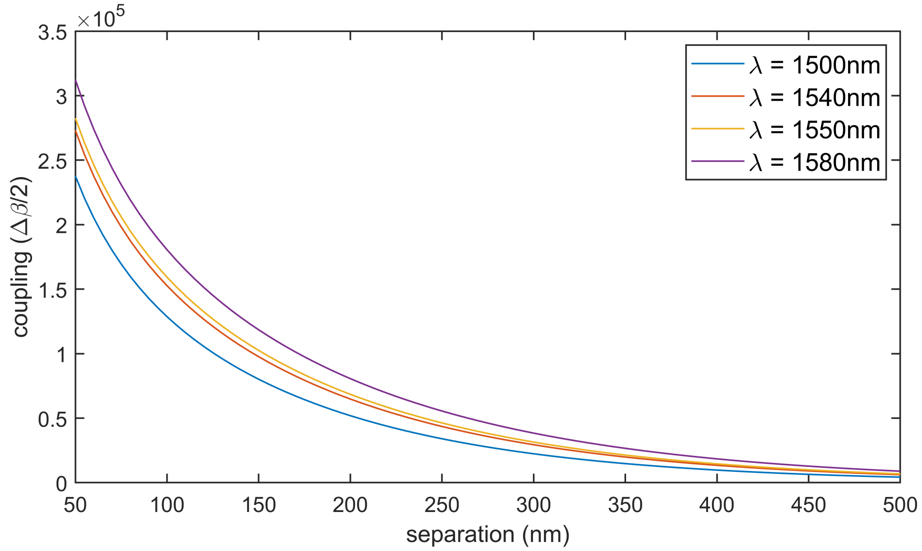

Fig. S1. (a) The coupling coefficients as a function of waveguide separation for different wavelengths.

Figure S3 shows the ratio of different coupling coefficients for different wavelengths. Essentially, these ratios define the array design as they determine the distances between adjacent waveguides. Here, the array is designed for operating at the central wavelength of $\lambda_0 = 1550\,\text{nm}$. According to this figure, changing the wavelength in the range of 1500 nm to 1580 nm results in only minor changes in the coupling ratios, which demonstrates the wideband functionality of the system.

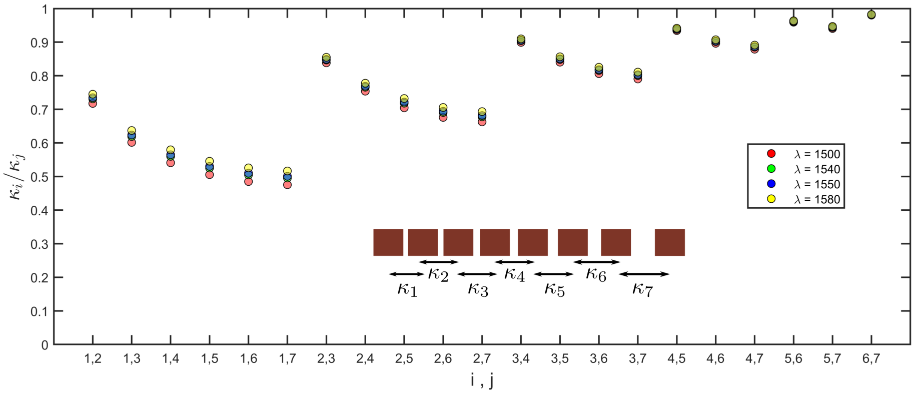

Fig. S2. **The ratios of the couplings of adjacent waveguides (κ) for the 15-waveguide array designed at the central wavelength $\lambda_0 = 1550\,\text{nm}$ and comparison with other wavelengths.**

2. SUPPLEMENTARY NOTE S2: LENSING FUNCTIONALITY OF THE STRUCTURE

To investigate the focusing property of the arrayed waveguide lens, we considered all input channels being excited uniformly and evaluated the evolution of the field in the AWL and at the output ports. We investigated this with coupled mode theory as well as finite element simulations. The following figure shows the results based on both approaches (left: coupled mode theory, right: finite element method). This figure clearly confirms the lensing property of the AWL.

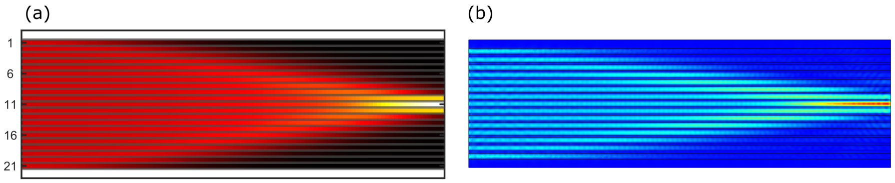

Fig. S3. **Lensing functionality of the coupled waveguide structure. (a) coupled-mode theory. (b) 2D full-wave simulations**
